# From validation to impact: the unfinished agenda of prehospital stroke care

**DOI:** 10.1055/s-0046-1823677

**Published:** 2026-07-16

**Authors:** Antonio Arauz

**Affiliations:** 1 Instituto Nacional de Neurología y Neurocirugía Manuel Velasco Suárez, Mexico City, Mexico.

Stroke remains one of the most time-critical neurological emergencies, where early recognition determines access to reperfusion therapies and ultimately functional outcomes. Contemporary guidelines from the American Heart Association and the European Stroke Organisation consistently emphasize that reducing prehospital delays is as critical as optimizing in-hospital workflows. 1 2 Despite advances in acute stroke management, including intravenous thrombolysis (IVT) and mechanical thrombectomy (MT), a substantial proportion of patients -particularly in low- and middle-income countries -do not receive timely treatment. 3

This gap reflects not only hospital-level limitations but, more importantly, failures occurring earlier in the chain of care. The prehospital phase is a decisive yet frequently underdeveloped component of stroke systems. Emergency medical services (EMS) are responsible for early recognition, triage, and routing of patients, and delays or inaccuracies at this stage can irreversibly compromise outcomes.

In this number of Arquivos de Neuro-Psiquiatria: Neurology and Neuroscience, Gorla et al. present the study *“Realistic simulation: construction and validation of scenarios for stroke recognition in prehospital care,”* offering a structured and methodologically rigorous contribution to the field. The authors developed and validated simulation-based scenarios aimed at improving stroke recognition in prehospital settings, employing a robust framework that integrates best practice recommendations and Bloom's taxonomy. Notably, they achieved unanimous agreement among expert judges, with a Content Validity Index of 1.0. The inclusion of both typical and atypical stroke presentations is particularly relevant, as diagnostic variability remains a major challenge in prehospital care. 4 Importantly, the study extends beyond theoretical development by implementing the scenarios in a real prehospital team. A noteworthy finding is that a significant proportion of participants reported insecurity when managing stroke patients, highlighting a critical gap in workforce preparedness that is likely generalizable across similar healthcare systems.

However, the central question is not whether these scenarios are valid, but whether they will translate into measurable improvements in stroke care. Content validation represents only an early step toward clinical impact. The absence of outcome evaluation -such as improvements in diagnostic accuracy, reductions in prehospital delays, or increased rates of reperfusion therapy -limits the ability to infer real-world effectiveness.

This limitation is particularly relevant in Latin America. Regional data have consistently shown that, despite a considerable proportion of patients arriving within the therapeutic window, only a minority receive reperfusion therapies. 3 This paradox reflects systemic inefficiencies in early recognition, triage, and coordination rather than solely resource constraints. In Mexico, national data consistently show that while a meaningful proportion of patients reach medical attention within the therapeutic window, the rates of IVT and MT remain disproportionately low. This was clearly demonstrated in the PREMIER registry, 5 where approximately 17% of patients arrived within the therapeutic window, yet fewer than 1% received IVT. 5 These findings underscore the critical role of prehospital systems as a key determinant of access to acute therapies.

In this context, simulation-based training should not be viewed as an isolated educational intervention, but as a strategic component of comprehensive stroke systems of care. When aligned with standardized prehospital scales, pre-notification protocols, and regional stroke networks, such training has the potential to improve diagnostic accuracy, streamline triage, and ultimately increase access to reperfusion therapies.

Another important consideration is scalability. Although the scenarios demonstrated high acceptance and realism, their implementation across diverse and resource-limited settings remains uncertain. Barriers related to infrastructure, personnel, and institutional prioritization may limit widespread adoption. Without integration into formal EMS training programs and national stroke strategies, even well-designed tools risk remaining underutilized.

Despite these challenges, the study provides a valuable contribution by addressing a clear and underexplored gap: the lack of validated, context-specific educational tools for prehospital stroke care. By demonstrating that it is feasible to construct and validate realistic simulation scenarios with high methodological rigor, the authors establish a foundation for future implementation and evaluation.

The next step is clear. Future research must move beyond validation to assess effectiveness, including impact on clinical performance, system-level metrics, and patient outcomes. Pragmatic studies, cost-effectiveness analyses, and integration into regional stroke networks will be essential to determine the true value of this approach.

Improving stroke outcomes requires more than technological innovation; it demands strengthening every link in the chain of care. The prehospital setting is where this chain most often fails -and where the greatest gains remain achievable.

The time for validation alone has passed. What stroke systems in Latin America now require is implementation, measurement, and accountability. Without these, even the most rigorously designed educational tools will remain academic exercises rather than instruments of change.

The work by Gorla et al. opens the door. It is now the responsibility of clinicians, educators, and health systems to walk through it and to ensure that earlier recognition translates into faster treatment and better outcomes for patients.
